# Tuning anhydrous proton conduction in single-ion polymers by crystalline ion channels

**DOI:** 10.1038/s41467-018-07503-4

**Published:** 2018-11-28

**Authors:** Onnuri Kim, Kyoungwook Kim, U. Hyeok Choi, Moon Jeong Park

**Affiliations:** 1 Department of Chemistry, Pohang University of Science and Technology (POSTECH), Pohang, 790-784 Korea; 2 Division of Advanced Materials Science, Pohang University of Science and Technology (POSTECH), Pohang, 790-784 Korea; 3 Department of of Polymer Engineering, Pukyong National University, Busan, 608-737 Korea

## Abstract

The synthesis of high-conductivity solid-state electrolyte materials with eliminated polarization loss is a great challenge. Here we show a promising potential of single-ion block copolymers with crystalline protogenic channels as efficient proton conductors. Through the self-organization of zwitterion, imidazole, and polystyrene sulfonate with controlled dipolar interactions therein, the distance between neighboring proton donors and acceptors in ionic crystals, as well as the dipolar orientation in nanoscale ionic phases was precisely tuned. This allowed a markedly high static dielectric constant comparable to water and fast structural diffusion of protons with a low potential barrier for single-ion polymers. The optimized sample exhibited a high proton diffusion coefficient of 2.4 × 10^–6 ^cm^2 ^s^–1^ under anhydrous conditions at 90 °C.

## Abstract (web summary)

High-conductivity solid-state electrolyte materials with minimal polarization loss are difficult to synthesize. Here the authors show single-ion block copolymers with crystalline protogenic channels having a promising potential to be used as efficient proton conductors.

## Introduction

In the past few decades, much effort has been devoted to the fabrication of proton-conducting materials^1,2^ ranging from protogenic polymers,^3−6^ liquid crystals,^7,8^ to porous organic frameworks.^9–11^ Although these studies were focused on different substances, they shared the same goal of achieving high proton conductivity via the creation of well-defined less tortuous ionic channels in mechanically stable supports.^2,12,13^ In addition, the suppression of anion diffusion in such materials has become increasingly important, since this diffusion is related to device polarization under applied dc voltage.^14^

The formation of well-defined ionic channels in mechanical supports can be achieved by covalent linking of ionophilic polymers to an ionophobic matrix,^15,16^ which results in spontaneous nanometer-scale self-assembly with adjustable geometry. Several studies have dealt with the design of microphase-separated ion-containing polymers of various architectures^13,17^ to establish morphology-transport relationship.

In addition to these efforts, much attention has been paid to the development of anhydrous proton conductors in view of the increased importance of achieving high proton conductivity at elevated temperatures.^17–22^ As part of this standpoint, much effort has been directed at the investigation of ionic liquid–containing polymers, which have been identified as promising next-generation ion conductors owing to the good thermal/chemical stability and high anhydrous conductivity at elevated temperatures.^17–26^

Although these studies have altered the stream of research on proton-conducting materials, the prevention of anion diffusion in ionic liquid–containing polymers remains a major challenge.^27^ Likewise, the same concern has been raised for other types of ionic conductors, i.e., metal-organic frameworks and covalent organic frameworks, owing to the substantial quantities of counter anions contained therein.^10,11^ Thus, although various high-conductivity materials have been developed, cases of their successful applications remain rare given that the actual current values observed during device operation are very low.^11,27^

The design of proton-conducting polymers in the form of so-called single-ion polymers would resolve the above issue, since the current is carried only by protons, and no anion depletion should theoretically be observed.^28^ However, compared to conventional polymer electrolytes, where both protons and counter anions exhibit mobility, single ion-polymers exhibit lower conductivities by orders of magnitude.^17–19,29,30^ With this in mind, most studies on single-ion polymers typically aim at expediting the relaxation of polymer chains and ions.^30,31^ The presence of crystalline phases in such materials, commonly formed by clustered ions (dimers, quadrupoles, and so on), is avoided because it tends to slow down polymer chain relaxation.

However, one needs to ask the question of whether the above reasoning is always correct. The apparent advantage of crystalline electrolytes is the gain of molecularly arranged ion-binding sites, which may favor the structural diffusion of protons over vehicular diffusion.^32^ Therefore, the formation of well-organized crystalline protogenic channels from single-ion conducting polymers should allow the fabrication of highly efficient proton-conducting materials featuring long-range hydrogen bonding networks.^33^

Herein, we report a peculiar type of proton conductors based on single-ion conducting block copolymers, where anion migration is fundamentally prevented. Notably, the self-organization of dipolar moieties in the above conductors results in the formation of ionic crystals confined in the nanoscale ionic phases of block copolymers, which allows us to control the distance between neighboring proton donors and acceptors, as well as the dipolar orientation in ionic phases to increase the rate of structural diffusion of protons along protogenic channels.

## Results

### Synthesis of single-ion conducting polymers

**Fig. 1:**
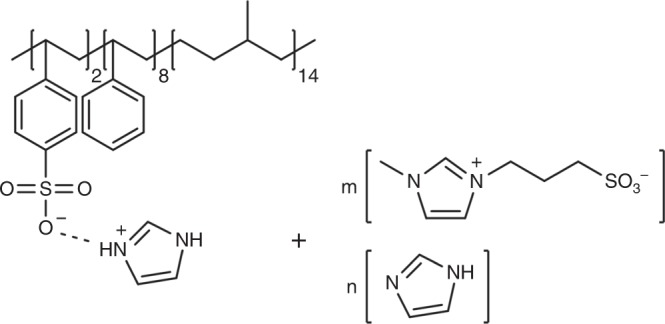
Molecular structures. Molecular structures of PSS:Im-*b*-PMB samples containing different molar ratios of ZImS:Im (1:0, 1:1, and 2:1 per mole of –SO_3_^–^-Im^+^ moieties in the polymer)

Poly(styrene-*b*-methylbutylene) (PS-*b*-PMB, 1.0-*b*-1.0 kg mol^–1^) block copolymer was synthesized as a precursor polymer. Degree of polymerization (*N*) of PS and PMB were 10 and 14, respectively. 20 mol.% of styrene units were randomly sulfonated to yield poly(styrene sulfonate-*b*-methylbutylene) (PSS-*b*-PMB, 1.2-*b*-1.0 kg mol^–1^) that was subsequently doped with one equivalent of imidazole (Im) relative to the quantity of sulfonic acid groups. The above sample was denoted as PSS:Im-*b*-PMB, and its chemical structure is shown in Fig. 1. PSS homopolymer with *N* = 10 and a sulfonation level of 20 mol.% was synthesized and doped by Im to prepare PSS:Im as a control sample.

In proton-conducting polymers such as PSS:Im-*b*-PMB and PSS:Im, the covalent attachment of anions to the polymer backbone eliminates anion migration. Considering the fact that most single-ion polymers suffer from low ionic conductivity, PSS:Im-*b*-PMB and PSS:Im were additionally modified by the introduction of a zwitterion, 3-(1-methyl-3-imidazolium) propanesulfonate (ZImS), and neutral Im to allow the modulation of ion dissociation degree and charge number density. The chemical similarities of Im and ZImS with PSS:Im ensure good thermodynamic compatibility with polymers. As denoted in Fig. 1, ZImS:Im molar ratio was varied as 1:0, 1:1, and 2:1 (per mole of –SO_3_^–^-Im^+^ moieties in the polymer), which were referred to as ZImS, ZImS/Im, and ZImS_2_/Im, respectively.

Glass transition temperature (*T*_g_) of PSS:Im in PSS:Im-*b*-PMB was 40 °C, which decreased to 34 °C, 29 °C, and 15 °C with the addition of ZImS, ZImS/Im, and ZImS_2_/Im, respectively (Supplementary Fig. 1). The low *T*_g_ value is the main reason for employing low molecular weight polymer in this study to achieve thermodynamic equilibrium without extensive thermal annealing.

### Structural analysis of PSS:Im-*b*-PMB comprising additives

Neat ZImS and neutral Im are commonly obtained as monoclinic crystals of the *P*2_1_*/c* space group.^34,35^ We found that the co-presence of ZImS and Im results in the formation of different crystal structures. For example, ZImS/Im afforded monoclinic crystals of the *P*1*m*1 space group (Supplementary Fig. 2 and Supplementary Table 1).

**Fig. 2:**
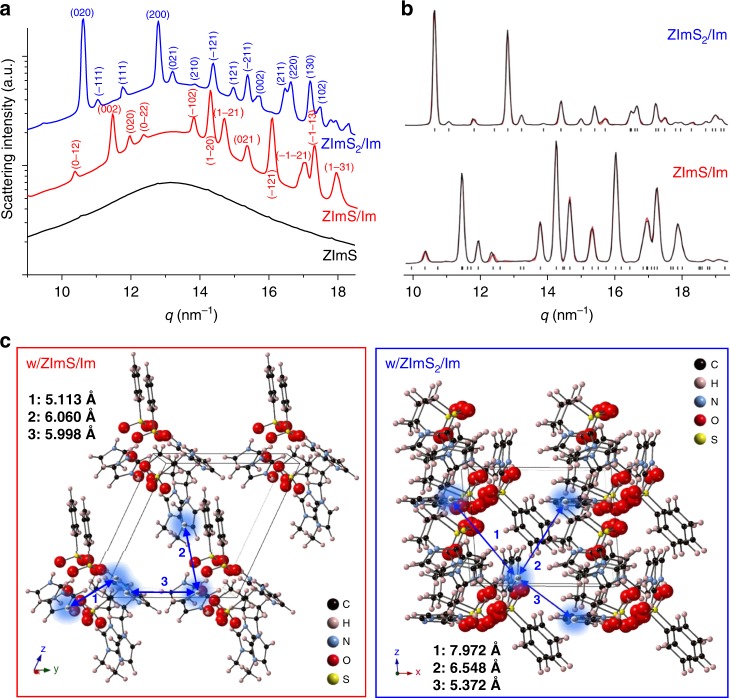
Self-organization of charged/dipolar moieties. **a** WAXS profiles of PSS:Im-*b*-PMB comprising various additives, measured at 25 °C, indexed with triclinic *P*1 space group (ZImS/Im) and monoclinic *P*2 1/*c* space group (ZImS_2_/Im). **b** Le Bail refinement of the WAXS patterns of PSS:Im-*b*-PMB with ZImS/Im and ZImS_2_/Im. **c** Molecular arrangements of SS:Im with ZImS/Im and ZImS_2_/Im. C, H, N, O, and S atoms are shown in black, pink, blue, red, and yellow, respectively. Distances between the neighboring Im^+^ and Im were marked in each crystal

The crystallization behavior of ZImS and Im was remarkably altered in the presence of polymer sulfonate groups. ZImS in PSS:Im phases did not crystallize when incorporated into PSS:Im-*b*-PMB, as confirmed by the wide-angle X-ray scattering (WAXS) profile shown in Fig. 2a. A typical amorphous halo, lacking the evidence of crystalline phases, was clearly revealed. On the contrary, the addition of ZImS/Im or ZImS_2_/Im resulted in the appearance of a series of diffraction peaks indexed to triclinic crystal of the *P*1 space group or monoclinic crystal of the *P*2 1/*c* space group, respectively. Miller indices of the reflection planes (*hkl*) of each crystal were obtained by Mercury DSD 3.9 software, as given in the figure.

Refinement of lattice parameters and structure factor amplitudes were obtained by Le Bail refinement of WAXS intensities and (*hkl*) Miller indices using a JANA2006 crystallographic program.^36^ Fig. 2b shows the refinement of data for ZImS/Im–containing and ZImS_2_/Im–containing PSS:Im-*b*-PMB and the results are summarized in Table 1. It is apparent that the lattice parameters of these crystals were broadly variable, depending on the ZImS:Im molar ratio.

**Table 1:** Ionic crystals formed in PSS:Im-*b*-PMB upon the addition of ZImS/Im and ZImS_2_/Im

PSS:Im-*b*-PMB	w/ ZImS/Im	w/ ZImS_2_/Im
Crystal structure	Triclinic	Monoclinic
Space group	*P* 1	*P* 2 1/*c*
*a* (Å) (α)	5.70 (62.5°)	9.82 (90.0°)
*b* (Å) (β)	11.93 (104.7°)	11.78 (94.9°)
*c* (Å) (γ)	12.66 (99.6°)	8.02 (90.0°)

To delineate how PSS:Im, ZImS, and neutral Im combined into crystalline phases, the conformations of Im-doped styrene sulfonate (SS:Im) with incorporated ZImS and neutral Im were optimized by ab initio calculations based on the density functional theory (DFT, B3LYP/6-31 G(d)) (Supplementary Fig. 3). Dissimilar dipolar orientations were expected for the samples depending on the molar ratio of ZImS:Im. Based on these structures and refined lattice parameters, the molecular arrangements of SS:Im, ZImS, and Im with different molar ratios are depicted in Fig. 2c.

While both crystals displayed an analogous distance of ~ 2.7 Å between Im^+^ and –SO_3_^–^ moieties in unit cells, the distances between the neighboring Im^+^/Im moieties were noticeably different, which is of importance for determining the structural diffusion of protons. For the sample comprising ZImS/Im, the short distances of 5.11 Å, 6.06 Å, and 5.99 Å were obtained in the *a-*, *b-*, and *c*-directions of the triclinic lattice. On the contrary, this distance increased to 5.37 Å, 7.97 Å, and 6.55 Å for the sample with ZImS_2_/Im showing the monoclinic lattice. Therefore, it was concluded that long-range proton conduction networks can be easily formed in PSS:Im phases comprising ZImS/Im.

It should be noted here that no evidence of crystal formation was observed for PSS:Im homopolymers with embedded ZImS, ZImS/Im, and ZImS_2_/Im (Supplementary Fig. 4), contrary to the cases of block copolymers described above. Thus, the confinement of dipolar moieties in nanoscale PSS domains was concluded to play a key role in facilitating crystal formation.

**Fig. 3:**
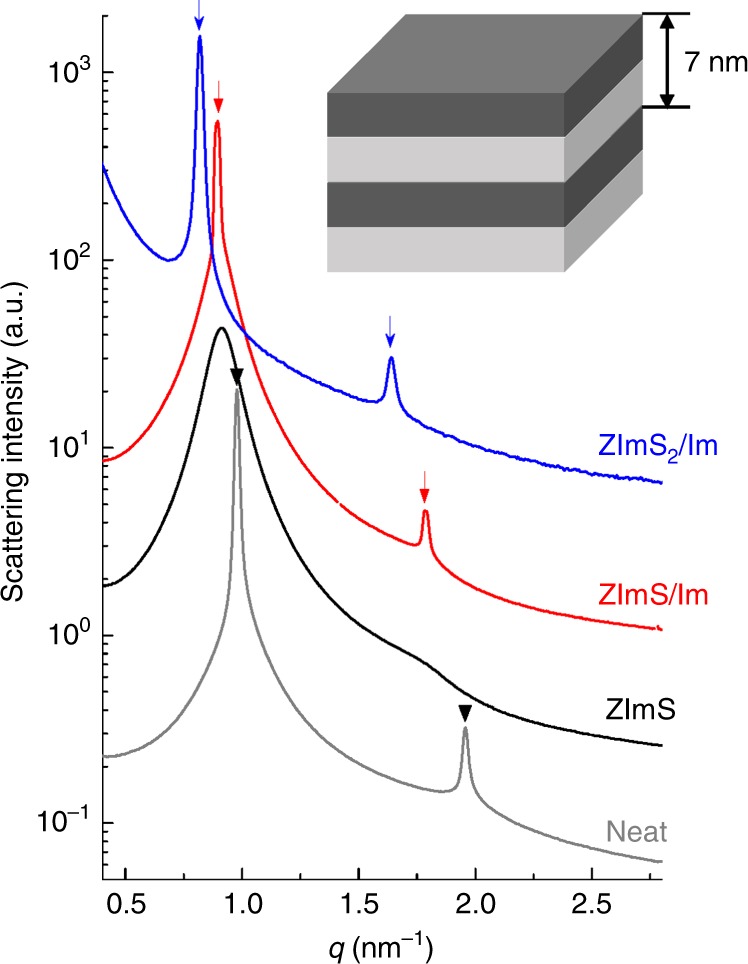
Effects of additives on self-assembly of PSS:Im-*b*-PMB. SAXS profiles of PSS:Im-*b*-PMB comprising various additives, measured at 25 °C. Bragg peaks at 1q*, 2q* (q* = 2π/*d*_100_) indicate lamellar morphology with ca. 7 nm domain size, as schematically depicted in the inset

The incorporation of ZImS and/or Im into PSS:Im-*b*-PMB affected its self-assembled morphology. Figure 3 shows representative small angle X-ray scattering (SAXS) profiles of the investigated samples, which were measured at room temperature and remained unchanged over the temperature range 25–130 °C. PSS:Im-*b*-PMB displayed lamellar morphology, as shown by the Bragg peaks (inverted black triangles) at 1q* and 2q* (q* = 2π/*d*_100_ and *d*_100_ = 6.4 nm). The presence of ZImS in PSS:Im phases of PSS:Im-*b*-PMB destabilized the microphase-separated morphology, which was attributed to the weakening of interactions between Im^+^ and SO_3_^-^ units of polymer by the presence of neighboring ZImS moieties. The well-defined lamellar structure was restored upon the introduction of ZImS/Im and ZImS_2_/Im, and both of these samples were shown to contain ionic crystals. The addition of ZImS, ZImS/Im, and ZImS_2_/Im into PSS:Im-*b*-PMB progressively increased the sample domain size to 6.9, 7.1, and 7.7 nm, respectively. The swelling of PSS:Im phases with additives can be envisaged by the reductions in the *T*_g_ value (Supplementary Fig. 1).

**Fig. 4:**
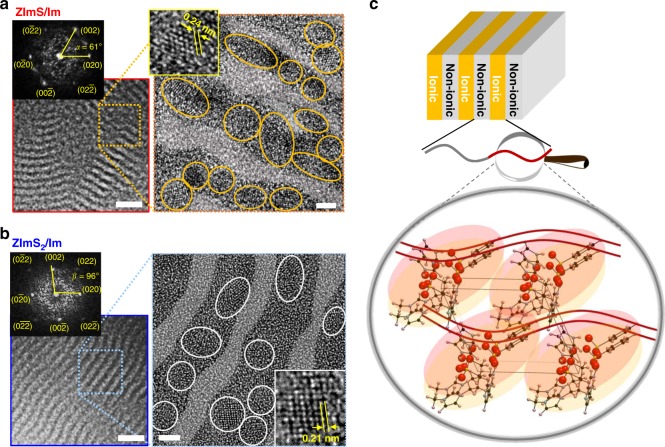
Ionic crystals in self-assembled lamellar grains. Bright-field TEM, high-resolution TEM micrographs, and FFT patterns obtained from PSS:Im-*b*-PMB with the addition of **a** ZImS/Im and **b** ZImS_2_/Im. The darkened area of lamellae indicates PSS:Im phases for both samples and ionic crystals are marked by circles in each TEM image for clarity. Scale bars in bright-field TEM and high-resolution TEM images are 20 nm and 2 nm, respectively. **c** Schematic illustration of the formation of ionic crystal in ionic phases of lamellar grains for PSS:Im-*b*-PMB with ZImS/Im

The size and location of ionic crystals were further examined by transmission electron microscopy (TEM) imaging. Figure 4a and Fig. 4b display representative bright-field TEM and high-resolution TEM micrographs of ZImS/Im– and ZImS_2_/Im–containing PSS:Im-*b*-PMB, respectively, revealing that 2–3 nm-sized crystalline structures were selectively confined within PSS:Im phases (darkened area) of lamellae for both samples. For clarity, ionic crystals are marked by circles in each high-resolution TEM image. The lattice parameters of ionic crystals obtained by fast Fourier transform (FFT) analysis were in good agreement with WAXS results.

Figure 4c schematically depicts the formation of ionic crystal in ionic phases of lamellar grains for ZImS/Im–containing PSS:Im-*b*-PMB, as a representative example. PS moieties are not shown for clarity. The crystal is formed by the co-organization of SS:Im, ZImS, and Im molecules in amorphous polymer matrix with controlled dipolar interactions therein.

### Proton transport properties

Is the formation of nano-confined ionic crystals with long-range hydrogen-bonding networks truly beneficial for the enhancement of structural diffusion of proton in single-ion conducting polymers? To answer this question, self-diffusion coefficients associated with N–H (*D*_N–H_) and C–H (*D*_C–H_) protons of Im^+^ and neutral Im were measured for PSS:Im-*b*-PMB with different additives using ^1^H pulsed-field gradient spin-echo (PGSE) NMR spectroscopy. Proton diffusion coefficient (*D*_*H*_^*+*^) was then calculated based on proton dissociation degree (*x*) of the samples, determined as 52% (with ZImS), 65% (with ZImS/Im), and 54% (with ZImS_2_/Im) by confocal Raman spectroscopy (Supplementary Fig. 5). The considerably high *x* value observed for PSS:Im-*b*-PMB with ZImS/Im is particularly noteworthy.

**Fig. 5:**
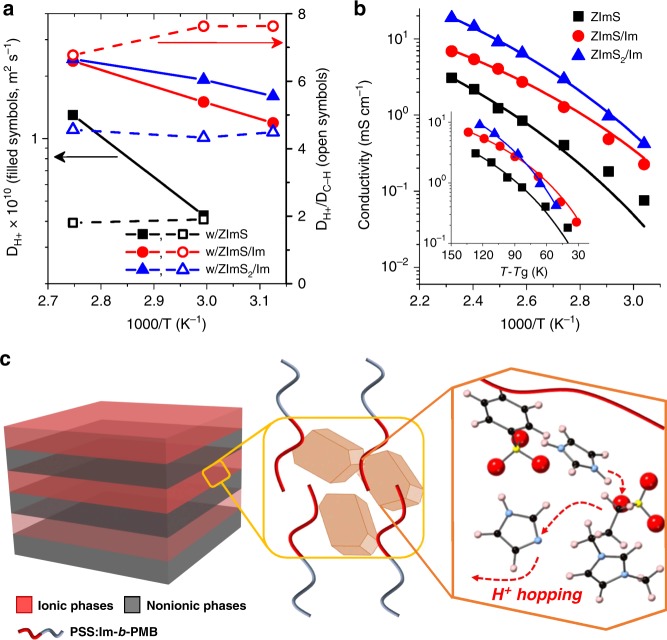
Proton transport properties. **a** Proton diffusion coefficients, **b** ionic conductivities, obtained for PSS:Im-*b*-PMB comprising additives, measured at different temperatures. In **a** and **b** the role of ionic crystals in determining the ion transport properties of single-ion polymers was clearly shown. **c** Schematics depicting dominant proton hopping mechanisms in PSS:Im-*b*-PMB containing ZImS/Im

Figure 5a shows the *D*_*H*_^*+*^ values and *D*_*H*_^*+*^ / *D*_*C-H*_ ratios of investigated samples, clearly displaying the role of ionic crystals in determining the proton diffusion behavior in single-ion polymers. Note that the accessible temperature window of the NMR spectrometer was 25–90 ^°^C because it was calibrated using ethylene glycol standard in water. Overall, *D*_*H*_^*+*^ values obtained for the sample comprising ZImS were lower than those obtained with ZImS/Im and ZImS_2_/Im by factors of 2–5. Notably, the *D*_*H*_^*+*^ / *D*_*C-H*_ ratio was as high as ~ 7.6 for the sample comprising ZImS/Im, whereas much lower values of 4.5 and 1.9 were obtained for ZImS_2_/Im-containing and ZImS-containing analogs, respectively. This finding suggests that proton hopping was most pronounced in the sample comprising ZImS/Im because of the shortest distances between protogenic moieties therein (Fig. 2c).

Figure 5b shows the temperature-dependent ionic conductivity of PSS:Im-*b*-PMB with different additives, revealing that this quantity decreased in the order of ZImS_2_/Im > ZImS/Im > ZImS, in good agreement with the results of diffusivity measurements. With excluded influence of *T*_g_ values of the samples on conductivity, as shown in inset plot of Fig. 5b, proton transport efficiency was highest for the ZImS/Im-containing sample if the sample temperature was lower than 90 °C. The lowest normalized conductivity was seen for the ZImS-embedded one for entire temperature window examined. Model fits by Vogel-Fulcher-Tammann equation (solid lines in Fig. 5b) further suggested dissimilar activation barriers to ion conduction, i.e., 1127 K for ZImS, 692 K for ZImS/Im, and 990 K for ZImS_2_/Im. The higher normalized conductivity and lower activation barrier obtained with ZImS/Im are notable.

Based on proton diffusion coefficients in Fig. 5a and ionic conductivities in Fig. 5b, Haven ratios were calculated for each sample at 60 °C and 90 °C. This is to quantify the dissimilar ion dissociation behavior of each sample by evaluating how much it deviates from unity. At 60 °C, the Haven ratios were 7.7, 7.1, and 5.2 for PSS:Im-*b*-PMB samples with ZImS, ZImS/Im and ZImS_2_/Im, respectively. Upon heating the samples to 90 ^°^C, at the temperature well-above the *T*_g_ values, the Haven ratios decreased to 6.2 (ZImS), 2.3 (ZImS/Im), and 1.2 (ZImS_2_/Im). Significant reductions in the Haven ratios seen only for crystal-forming samples are particularly noteworthy, indicating high dissociation degree of ions bound in the crystal structure to contribute to efficient proton transport, as schematically illustrated in Fig. 5c. Note in passing that the grain size of lamellar structures decreased in the order of ZImS_2_/Im > ZImS/Im > ZImS. This would also have an impact on the Haven ratio, but the concrete underpinning remains as a future study.

It is worth noting that we confirmed no substantial effects of the molecular weight of PSS:Im-*b*-PMB on ionic crystal formation and conductivity enhancement. Supplementary Fig. 6 shows scattering profiles and temperature-dependent conductivity data obtained from PSS:Im-*b*-PMB (20–39 kg mol^−1^, a sulfonation level of 25 mol.%, lamellae, *d*_100 _=56 nm) with additives. Ionic crystals were again formed only with ZImS/Im and ZImS_2_/Im, leading to markedly enhanced ionic conductivity, compared with amorphous sample comprising ZImS. This finding suggests the crucial role of co-organization of neutral Im and ZImS in developing the unique crystalline structures in PSS:Im phases of PSS:Im-*b*-PMB, which is intimately related to the improved ion transport properties.

Recent work of Winey and coworkers^37^ may be relevant to our results given that ionic moieties (–SO_3_^-^–H_3_O^+^) are confined by surrounding crystalline matrix (polyethylene backbones). However, the fundamental difference lies in the fact that PSS:Im-*b*-PMB block copolymers have essentially amorphous PS backbones and PSS chains directly involved in the ionic crystal formation for participation in proton transport.

## Discussion

**Fig. 6:**
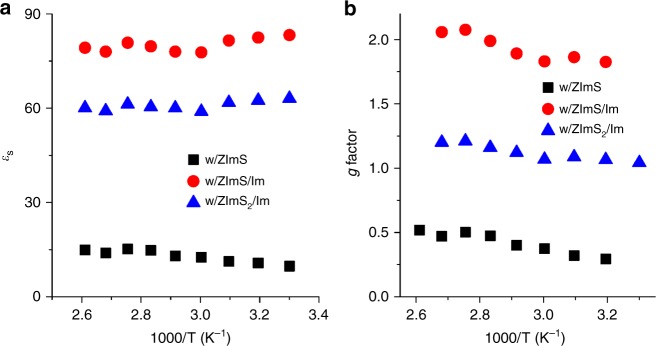
Dielectric properties. **a** Static dielectric constant and **b** Kirkwood-Fröhlich *g* factor obtained for PSS:Im-*b*-PMB comprising additives, measured at different temperatures

We explored the dielectric relaxation behavior of the abovementioned samples. Figure 6a shows static dielectric constant (*ε*_s_) of the samples, measured at different temperatures (dielectric permittivity spectra are given in Supplementary Fig. 7). ZImS/Im–containing PSS:Im-*b*-PMB exhibited high *ε*_s_ values over 78 while the lowest *ε*_s_ value of 13 was observed with the incorporation of ZImS. It should be noted that *ε*_s_ value of neat PSS:Im-*b*-PMB was as low as 5.

The origin of the differences in *ε*_s_ values was elucidated by analyzing local directional correlations between neighboring dipoles for each sample. Based on DFT calculations, we determined three major dipole moments of ion pair (SO_3_^-^-Im^+^, 7 D), ZImS (17.5 D), and neutral pair (Im-ZImS, 19.3 D), as listed in Supplementary Table 2. This computational method also enabled us to estimate the optimized dipole structures, as provided in Supplementary Fig. 3. The Kirkwood-Fröhlich *g*-factor was calculated by equation (1)^30^ using the number density of dipolar moieties (*v*_*i*_) and the dipole moment (*m*_*i*_) (listed in Supplementary Table 2). The overlap of dipole polarizability volumes, defined as the ratio of Debye’s polarizability volume *V*_p_ to molecular volume *V*_m_, was calculated by equation (2). Representative *V*_p_/*V*_m_ values of each sample obtained at 60 °C are listed in Supplementary Table 2.

1 $$g = {\frac{{9\varepsilon _0kT}}{{\mathop {\sum}\nolimits_i {v_im_i^2}}}{\frac{{\left( {\varepsilon _{\mathrm{s}} - \varepsilon _\infty } \right)\left( {2\varepsilon _{\mathrm{s}} + \varepsilon _\infty } \right)}}{{\varepsilon _{\mathrm{s}}\left( {\varepsilon _\infty + 2} \right)^2} }}}$$

2 $$\frac{{V_{\mathrm{p}}}}{{V_{\mathrm{m}}}} = \mathop {\sum }\limits_i \frac{{v_im_i^2}}{{12\pi \varepsilon _0kT}}$$

Figure 6b illustrates the temperature dependence of the *g*-factor, showing that ionic orientational polarization is present in all samples owing to the presence of SO_3_^–^–Im^+^ ion pairs. With ZImS addition, the low *g*-factor of < 1 and *V*_p_/*V*_m_ < 1 were obtained, which indicated that the ion pair (SO_3_^–^–Im^+^) and ZImS tend to form a quadrupole (i.e., two dipoles are antiparallel each other), leading to offset of dipole moment by dipole-dipole attraction. This is consistent with the lowest *ε*_s_ value of PSS:Im-*b*-PMB containing ZImS (Fig. 6a).

On the contrary, the highest *g*-factor of ~ 2 was obtained for PSS:Im-*b*-PMB comprising ZImS/Im to enhance the *ε*_s_ value. This can be rationalized by the DFT calculations (Supplementary Fig. 3), displaying that neutral Im breaks the quadrupole to make surrounding dipoles are arranged parallelly, thereby providing the system with a net dipole moment. The net dipole moment is intimately connected to close packing of neighboring dipolar moieties and the dipole-dipole interaction acts as an enthalpic contribution to the ionic crystal formation. This intriguing dipolar orientation was further envisaged by the *V*_p_/*V*_m_ ratio of ~1 (Supplementary Table 2), signaling that ion pairs were forced to strongly interact at the overlap point.^30^

The sample with ZImS_2_/Im exhibited *g* ≈ 1 and *V*_p_/*V*_m_ > 1, ascribed to the increased density of dipoles, resulting in a significant dipole overlap to restrict dipolar correlation. This leads us to conclude that the dipole correlation affects the structure (amorphous vs. crystalline) and proton hopping distance (ordered dipolar moieties reduce the distance).

It is worth noting that no substantial enhancement of *ε*_s_ was observed for PSS:Im homopolymers with various additives (Supplementary Fig. 8). This finding implies that synergistic dipole alignments occurred only in samples containing nano-confined ionic crystals, the formation of which is thus of key importance for improving proton transport properties.

We would like to conclude this paper by commenting on the single-ion properties of PSS:Im-*b*-PMB comprising additives. Because zwitterion is composed of covalently connected cation and anion, it is electroneutral. Nevertheless, the excessive use of zwitterion in polymer electrolytes may result in charge depletion at electrolyte/electrode interfaces at a given voltage. However, this was avoided in our polymers because the ZImS is packed in ionic crystals and further confined within PSS:Im domains, as confirmed by polarization experiments (Supplementary Fig. 9). Note that the content of ZImS is low as 13 wt% for PSS:Im-b-PMB containing ZImS/Im (neutral Im content is 4 wt%).

Although a large number of ion-containing polymers have been reported in literature, cases of developing well-defined protogenic crystals with in-depth underpinning of conductivity-structure relationship remains rare. Given that strong dipolar interactions and effective confinements of ionic moieties in polymer matrix can inhibit leaching of constituents over time, our polymers have potential advantages over conventional ionic liquid-containing polymer electrolytes. Our approach should find wide applicability in various electrochemical applications.

In summary, we demonstrate that proton transport rate in single-ion conducting polymers can be enhanced by creating high-dielectric-constant crystalline protogenic channels in an amorphous polymer matrix. Specifically, the strong dipolar interactions in nanoscale ionic phases of block copolymers resulted in the formation of 2–3 nm-sized ionic crystals packed within. The developed approach allowed the realization of dominant structural diffusion of protons, improved ion dissociation degree, and significantly enhanced dielectric constant of the single-ion polymers.

## Method

### Synthesis of sulfonated polymers

A poly(styrene-*b*-methylbutylene) (PS-*b*-PMB, 1.0-*b*-1.0 kg mol^-1^) precursor block copolymer was synthesized by sequential anionic polymerization of styrene (ReagentPlus®, 99.9%, Sigma-Aldrich) and isoprene ( ≥ 99%, Sigma-Aldrich) using sec-butyllithium initiator in cyclohexane, followed by selective hydrogenation of isoprene units using Ni-Al catalyst. A polystyrene (PS, 1.0 kg mol^-1^) homopolymer was also prepared by anionic polymerization as a control sample. The absolute molecular weights of PS-*b*-PMB block copolymer and PS homopolymer were characterized by end-group analysis using ^1^H Nuclear Magnetic Resonance (^1^H-NMR, Bruker AVB-300) spectroscopy in CDCl_3_. Size exclusion chromatography (SEC, Waters Breeze 2 HPLC) was used to characterize polydispersity indices of PS-*b*-PMB and PS, which were determined to be 1.03 and 1.02, respectively, with PS standards and tetrahydrofuran (THF) eluent. Subsequent sulfonation reaction of PS-*b*-PMB block copolymer and PS homopolymer using acetic sulfate in anhydrous dichloromethane ( ≥ 99.8%, Sigma-Aldrich) yielded PSS-*b*-PMB block copolymer and PSS homopolymer. The sulfonation level was determined to be 20 mol.% by ^1^H-NMR in acetone-*d*_6_. The PSS-*b*-PMB and PSS were doped with equivalent imidazole ( ≥ 99%, Sigma-Aldrich) to the mole of sulfonic acid group of polymer using methanol to yield PSS:Im-*b*-PMB block copolymer and PSS:Im homopolymer.

### Synthesis of zwitterion

3-(1-methyl-3-imidazolium) propanesulfonate (ZImS) was synthesized by mixing 1-methylimidazole (ReagentPlus®, 99.9%, Sigma-Aldrich) and 1,3-propanesultone ( ≥ 99%, Sigma-Aldrich) in acetone ( ≥ 99.5%, Sigma-Aldrich) at room temperature. After 5 days of stirring, the mixtures were purified by repeated filtration and precipitation. White powder was recovered by vacuum drying at 60 °C and the successful synthesis of ZImS was confirmed by combining ^1^H-NMR and Fourier transform Infrared (FT-IR, a Spectrum Two, PerkinElmer, USA) spectroscopy experiments.

### Preparation of single-ion conducting polymers

Inhibitor-free anhydrous THF ( ≥ 99.9%) and anhydrous methanol (99.8%) were purchased from Sigma-Aldrich and used without further purification. Predetermined quantities of PSS:Im-*b*-PMB (or PSS:Im), ZImS, and neutral Im were dissolved into 80/20 vol.% methanol/THF mixtures to prepare ca. 5 wt% solutions using the balance (Mettler Toledo, ME 204) inside the argon-filled glove box. Under argon atmosphere, solvents were slowly evaporated at room temperature for 2 days and were further exposed to vacuum at 70 °C for 7 days.

### X-ray scattering (SAXS and WAXS) experiments

Synchrotron SAXS and WAXS measurements on the single-ion polymers were performed using the PLS-II 4 C and 9 A beamlines at the Pohang Accelerator Laboratory (PAL), equipped with temperature-controlled sample stage and two-dimensional detector. The samples were laminated into an air-tight cell inside argon-filled glove box to avoid the issue of water contamination. Three different sample-to-detector distances of 2.0 m, 0.2 m, and 0.1 m were used to cover a wide scattering wave vector *q* (*q* = 4*π* sin(*θ*/2)/*λ*, where *θ* is the scattering angle). The scattering data were azimuthally averaged to obtain intensity versus *q*.

### Transmission electron microscopy (TEM) experiments

The self-assembled morphologies and crystalline structures of PSS:Im-*b*-PMB comprising ZImS/Im and ZImS_2_/Im were examined by bright-field TEM and high-resolution TEM experiments (JEOL JEM-2200FS). The samples were cryo-microtomed at −120 °C to obtain thin sections with thicknesses of ca. 100 nm using a RMC Boeckeler PT-XL Ultramicrotome. For bright-field TEM imaging, the PSS:Im phases were darkened by ruthenium tetroxide (RuO_4_) staining.

### Fourier transform Raman (FT-Raman) experiments

Confocal Raman spectra of PSS:Im-*b*-PMB comprising additives were measured using a WITEC Alpha 300 R Raman spectroscope (WITec, Ulm, Germany), equipped with a HeNe laser, under low laser excitation power below 3 mW. The spatial resolution of the spectrometer was 250 nm. The samples were placed on dimple-patterned slide glasses inside glovebox and covered with a window glass to avoid the contact of samples with air.

### Self-diffusion coefficient measurements

Pulsed gradient spin echo (PGSE) ^1^H-NMR experiments on PSS:Im-*b*-PMB block copolymers comprising various additives were performed using a Bruker AVB-300 spectrometer. The samples were loaded into 4 mm (o.d.) NMR microtubes in an argon-filled glovebox and were sealed with caps. Time interval of the field gradient and duration time between two gradient pulses were in the range 1–10 ms and 0.1–0.3 s, respectively. The samples were equilibrated for 1 h at each temperature prior to the measurements, which was calibrated using ethylene glycol standard.

### Ionic conductivity measurements

The through-plane conductivities of PSS:Im-*b*-PMB comprising additives were measured by impedance spectroscopy using PARSTAT 2273 and Solartron 1260 A. The dimension of solvent-cast membrane was 0.8 cm × 0.8 cm × 200 μm, which was placed inside engraved counter Pt electrode and covered by Pt working electrode with Kapton spacers inside the glove box. Samples were annealed for 30 min at each temperature and data were collected in a wide frequency range of 10–10^5^ Hz.

### Dielectric relaxation spectroscopy

The dielectric permittivity spectra of the samples (the same samples employed for impedance spectroscopy measurements) were acquired using a Novocontrol GmbH Concept 40 broadband dielectric spectrometer, equipped with Novocontrol sample chamber, under a sinusoidal voltage of 0.1 V in a frequency range of 10–10^7^ Hz. The sample cell consists of two polished brass electrodes (10 mm-diameter top electrode and 30 mm diameter bottom electrode) and a silica spacer. Samples were annealed at 120 °C for 1 h prior to the measurements to achieve good contact of the sample with electrodes.

## Electronic supplementary material

Supplementary Information

## Data Availability

The data supporting the findings of this study are available within the paper and its Supplementary Information. Other datasets analyzed for the present study are available from the corresponding author on reasonable request.
