# Supplementary material for: Tuning anhydrous proton conduction in single-ion polymers by crystalline ion channels
Source: Nat Commun. 2018 Nov 28;9:5029. doi: 10.1038/s41467-018-07503-4 (PMC6261987; doi:10.1038/s41467-018-07503-4)
Supplement: Supplementary file 1 — Supplementary Information [file 41467_2018_7503_MOESM1_ESM.pdf]

**Supplementary Information**

# Tuning Anhydrous Proton Conduction in Single-Ion Polymers by Crystalline Ion Channels

Onnuri Kim[1§], Kyoungwook Kim[2§], U Hyeok Choi[3], and Moon Jeong Park[1,2*]

[1]Department of Chemistry, [2]Division of Advanced Materials Science, Pohang University of Science and Technology (POSTECH), Pohang, Korea 790-784,

[3]Department of Polymer Engineering, Pukyong National University, Busan, Korea 608-737.

[§] These two authors contributed equally.

* Corresponding author

(moonpark@postech.ac.kr)

**Supplementary Figures**

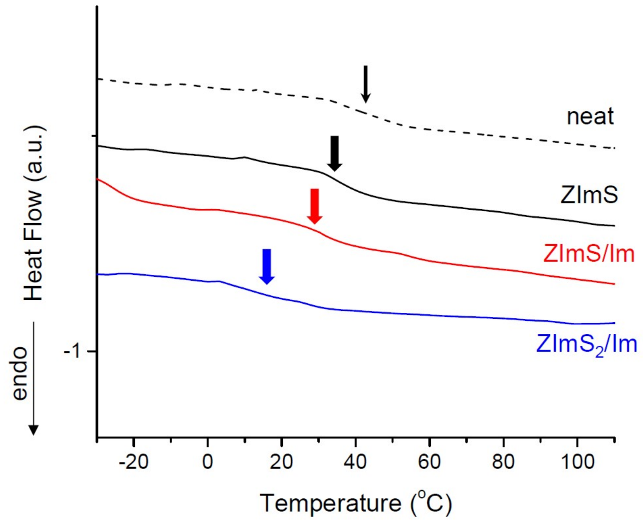


**Supplementary Figure 1. Thermal transitions of PSS:Im-*b*-PMB with additives.** DSC thermograms of PSS:Im-*b*-PMB (1.2-*b*-1.0 kg mol$^{-1}$) containing ZImS, ZImS/Im, and $ZImS_2$/Im, compared with that of neat PSS:Im-*b*-PMB, indicating a decrease in the $T_g$ value of PSS:Im with additives. $T_g$ of PMB (-68 °C) was not affected by additives.

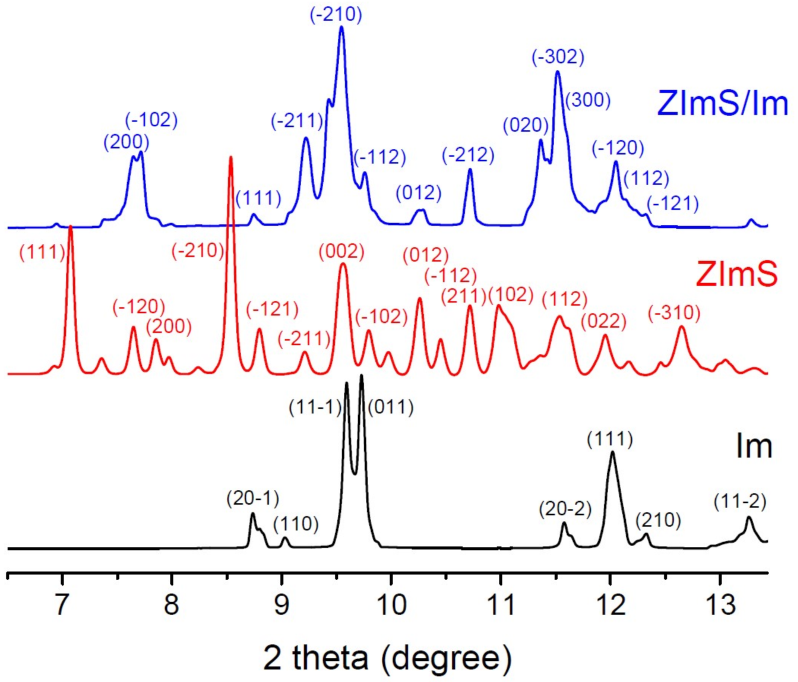


**Supplementary Figure 2. Co-organization of ZImS and Im.** WAXS profiles of neat Im, and neat ZImS, and ZImS/Im. Miller indices of the reflection planes (*hkl*) of each crystal were obtained by Mercury DSD 3.9 software, as given in the figure.

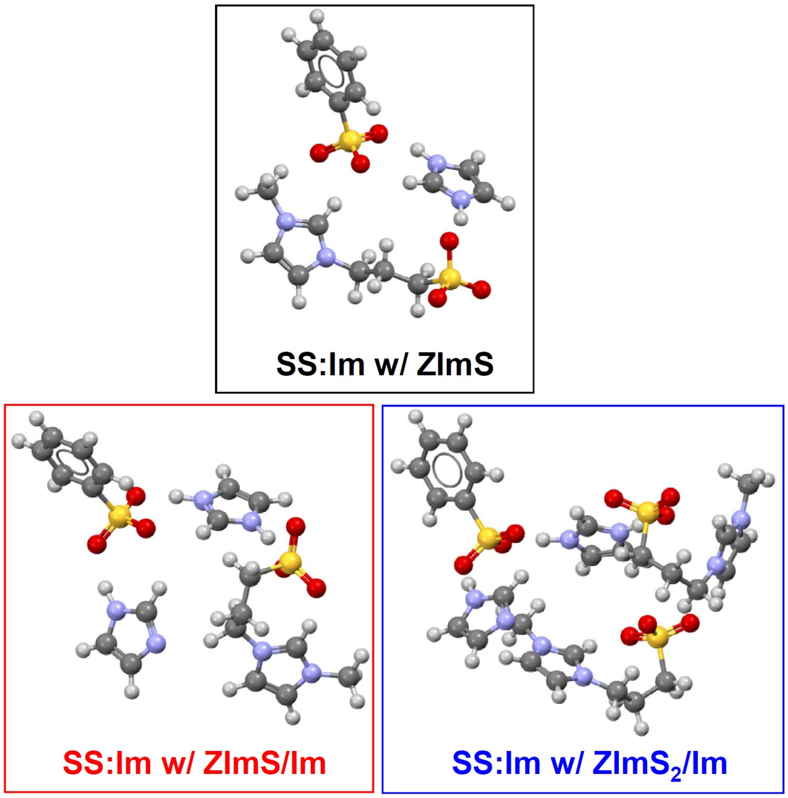


**Supplementary Figure 3. Dissimilar dipolar orientations.** Molecular conformations of SS:Im with incorporated ZImS and neutral Im, for which the geometries had been optimized by *ab initio* calculations based on the density functional theory (B3LYP/6-31G(d)).

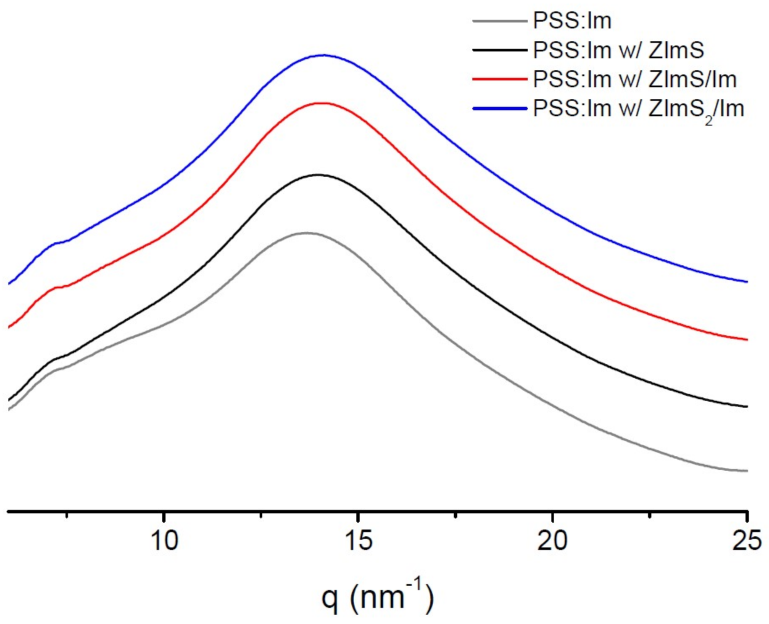


**Supplementary Figure 4. Amorphous characteristics of PSS:Im homopolymers.** WAXS profiles of PSS:Im homopolymers (1.2 kg mol$^{-1}$ and sulfonation level of 20 mol.%) comprising ZImS, ZImS/Im, and ZImS$_2$/Im, compared with that of neat PSS:Im, measured at 25 °C. It is evident that all samples have amorphous ionic phases.

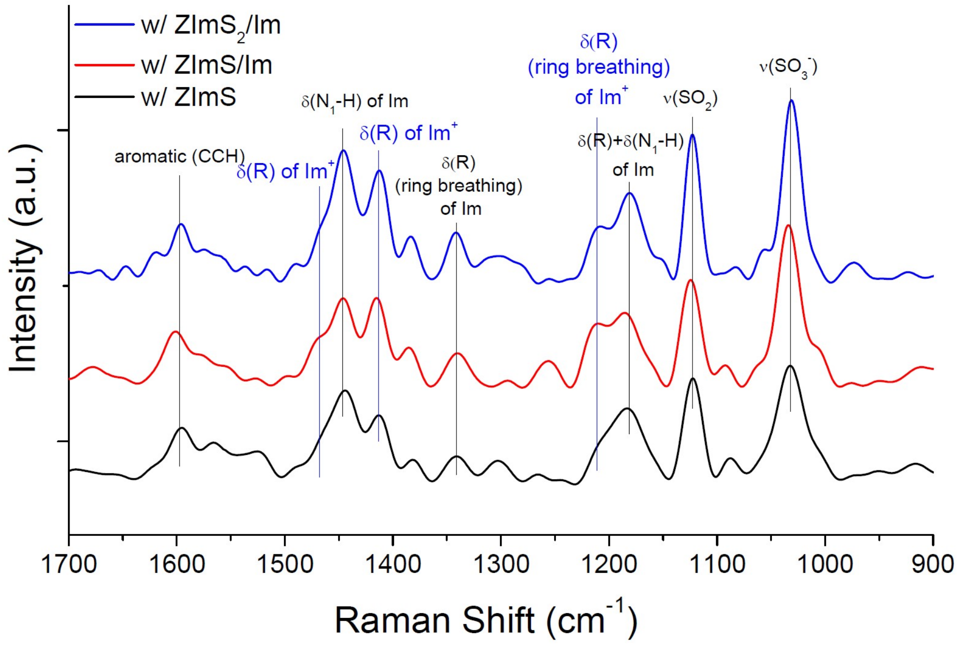


**Supplementary Figure 5. Dissimilar proton dissociation degrees.** Fourier transform Raman spectra of PSS:Im-*b*-PMB comprising ZImS, ZImS/Im, and ZImS$_2$/Im over the wavenumber ranges 900 – 1700 $cm^{-1}$. C–CH aromatic ring quadrant of PSS chains (at 1600 $cm^{-1}$) was used as an internal reference. As assigned in the figure, the absorption band at 1210 $cm^{-1}$ indicates the ring breathing of $Im^+$, whereas that of neutral Im is shown at 1338 $cm^{-1}$. The peak at 1413 $cm^{-1}$ signifies $CH_3$-N and $CH_2$-N stretching of $Im^+$, whereas that at 1446 $cm^{-1}$ denotes the N-H stretching of neutral Im. From the intensity ratio, e.g., $I^{1210}/(I^{1210} + I^{1338})$, the degree of proton dissociation was calculated as 52%, 65%, and 54% for the samples containing ZImS, ZImS/Im, and ZImS$_2$/Im, respectively.

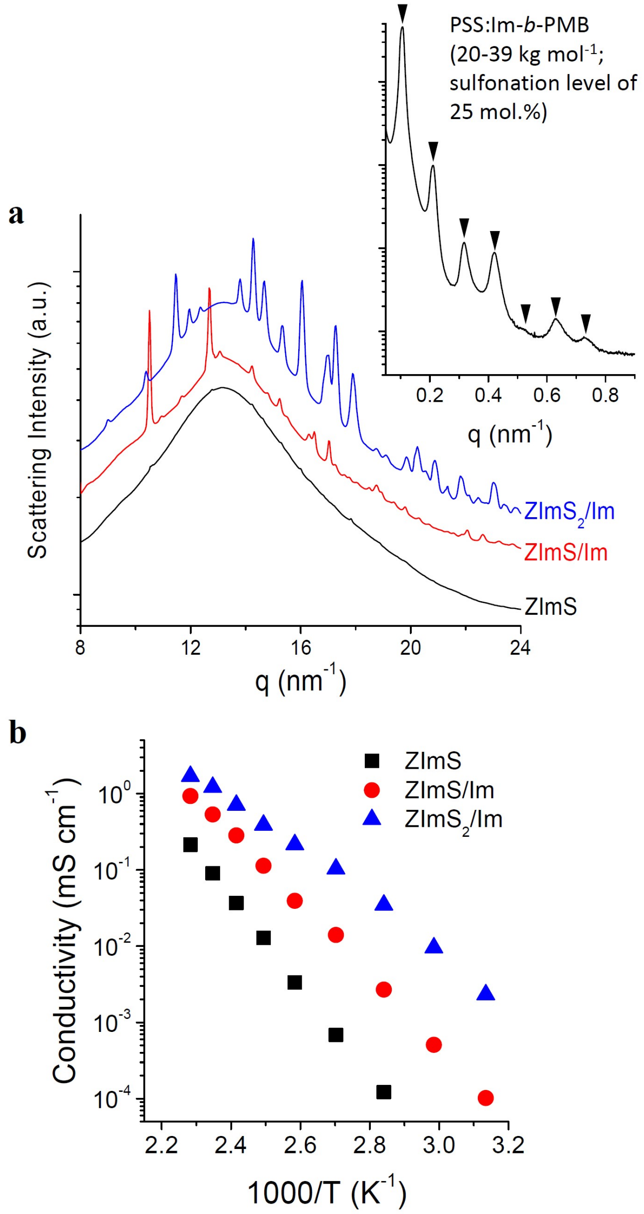


**Supplementary Figure 6. High molecular weight PSS:Im-*b*-PMB comprising additives.** **a**, WAXS profiles of PSS:Im-*b*-PMB (20-39 kg mol$^{-1}$ and a sulfonation level of 25 mol.%) comprising ZImS, ZImS/Im, and ZImS$_2$/Im, measured at 25 °C. SAXS profile of neat PSS:Im-*b*-PMB displaying lamellar morphology ($d_{100}$ = 56 nm) is shown in the inset. **b**, Temperature-dependent ionic conductivity of PSS:Im-*b*-PMB (20-39 kg mol$^{-1}$) comprising additives.

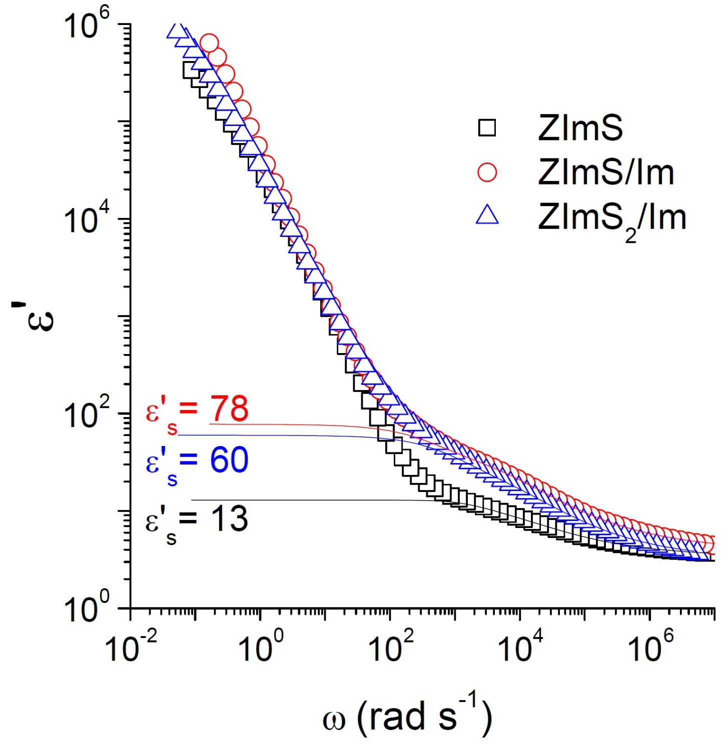


**Supplementary Figure 7. Dielectric relaxation behavior.** Representative dielectric permittivity spectra of PSS:Im-*b*-PMB copolymers with various additives, measured at 60 °C. The spectra with model fits were horizontally shifted to show the static dielectric constants, $\varepsilon_s$.

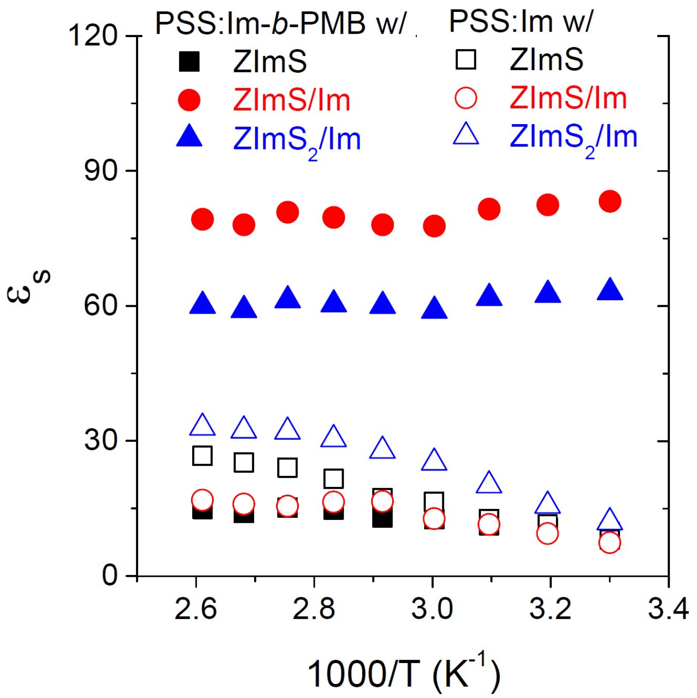


**Supplementary Figure 8. Dissimilar dipolar arrangements.** The static dielectric constants, $\varepsilon_s$, of PSS:Im-*b*-PMB and PSS:Im with the addition of ZImS, ZImS/Im, and $ZImS_2$/Im at different temperatures, revealing dissimilar dipolar arrangements in ionic phases of block copolymers and homopolymers.

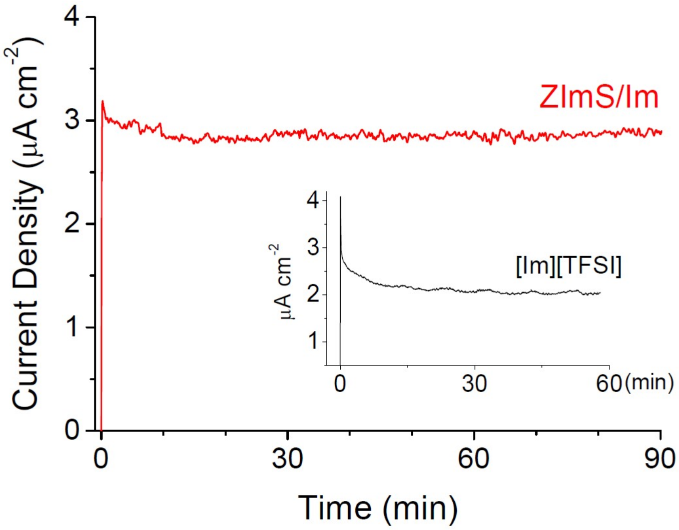


**Supplementary Figure 9. Polarization experiments.** Current density profile of PSS:Im-*b*-PMB containing ZImS/Im, after polarizing the sample with $\Delta V$ of 100 mV. Symmetric cell was employed by sandwiching the polymer with carbon electrodes. PSS:Im-*b*-PMB containing ionic liquid was used as a control.

**Supplementary Tables**

**Supplementary Table 1. Crystal Structures and Lattice Parameters of Neat Im, Neat ZImS, and ZImS/Im Mixture**

| | Im | ZImS | ZImS/Im |
|---|---|---|---|
| Crystal structure | Monoclinic | Monoclinic | Monoclinic |
| Space group | $P2_1$/c | $P2_1$/c | *P*1m1 |
| *a* (Å) (α) | 9.8 (90°) | 9.8 (90°) | 11.1 (90°) |
| *b* (Å) (β) | 5.5 (117°) | 11.4 (98°) | 7.0 (113.0°) |
| *c* (Å) (γ) | 7.8 (90°) | 8.1 (90°) | 10.1 (90°) |

**Supplementary Table 2. Dielectric Relaxation Parameters of PSS:Im-*b*-PMB Block Copolymers Comprising ZImS, ZImS/Im, and ZImS$_2$/Im**

| PSS:Im-*b*-PMB | Dipolar moiety | Number density[a] ($v$, $nm^{-3}$) | Dipole moment[b] ($m$, D) | g[c] | $V_p/V_m$[d] |
|---|---|---|---|---|---|
| w/ ZImS | Ion pair ($SO_3^- – Im^+$) | 0.36 | 7 | 0.4 | 0.8 |
| | ZImS | 0.32 | 17.5 | | |
| w/ ZImS/Im | Ion pair ($SO_3^- – Im^+$) | 0.32 | 7 | 1.9 | 1.1 |
| | Neutral pair (Im - ZImS) | 0.39 | 19.3 | | |
| w/ ZImS$_2$/Im | Ion pair ($SO_3^- – Im^+$) | 0.27 | 7 | 1.1 | 1.5 |
| | Neutral pair (Im - ZImS) | 0.32 | 19.3 | | |
| | ZImS | 0.24 | 17.5 | | |

[a]estimated from the group contribution method based on molecular structure.

[b]calculated using a DFT exchange-correlation functional, *B3LYP*.

[c]calculated using eq (1) at 343 *K*

[d]calculated using eq (2) at 343 *K*
